# 2-(Bicyclo­[2.2.1]hept-5-en-2-yl)-1*H*-pyrrolo­[2,3-*b*]pyridine

**DOI:** 10.1107/S1600536810022087

**Published:** 2010-06-26

**Authors:** Roland Selig, Dieter Schollmeyer, Wolfgang Albrecht, Stefan Laufer

**Affiliations:** a Eberhard-Karls-University Tübingen, Auf der Morgenstelle 8, 72076 Tübingen, Germany; b University Mainz, Duesbergweg 10-14, 55099 Mainz, Germany; c c-a-i- r biosciences GmbH, Paul-Ehrlich-Strasse 15, 72076 Tübingen, Germany

## Abstract

The crystal structure of the title compound, C_14_H_14_N_2_, displays inter­molecular N—H⋯N hydrogen bonds, forming dimers of enanti­omeric mol­ecules *via* a crystallographic centre of inversion.

## Related literature

For the general synthetic procedure for 2-substituted 7-aza­indoles, see Davis *et al.* (1992 ▶). 
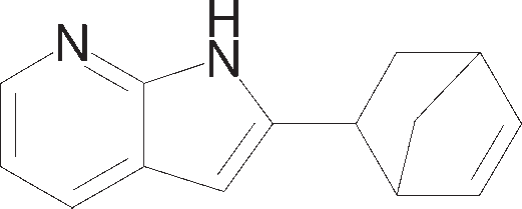


## Experimental

### 

#### Crystal data

C_14_H_14_N_2_
*M* *_r_* = 210.27
Monoclinic, 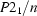

*a* = 7.7837 (12) Å
*b* = 8.9867 (14) Å
*c* = 15.973 (3) Å
β = 96.408 (8)°
*V* = 1110.3 (3) Å^3^
*Z* = 4
Cu *K*α radiation
μ = 0.58 mm^−1^
*T* = 193 K
0.40 × 0.30 × 0.20 mm

#### Data collection

Enraf–Nonius CAD-4 diffractometer
2274 measured reflections
2113 independent reflections
1940 reflections with *I* > 2σ(*I*)
*R*_int_ = 0.050
3 standard reflections every 60 min  intensity decay: 2%

#### Refinement

*R*[*F* ^2^ > 2σ(*F* ^2^)] = 0.082
*wR*(*F* ^2^) = 0.227
*S* = 1.09
2113 reflections
145 parameters
H-atom parameters constrained
Δρ_max_ = 0.47 e Å^−3^
Δρ_min_ = −0.36 e Å^−3^

### 

Data collection: *CAD-4 Software* (Enraf–Nonius, 1989 ▶); cell refinement: *CAD-4 Software*; data reduction: *CORINC* (Dräger & Gattow, 1971 ▶); program(s) used to solve structure: *SIR97* (Altomare *et al.*, 1999 ▶); program(s) used to refine structure: *SHELXL97* (Sheldrick, 2008 ▶); molecular graphics: *PLATON* (Spek, 2009 ▶); software used to prepare material for publication: *PLATON*.

## Supplementary Material

Crystal structure: contains datablocks I, global. DOI: 10.1107/S1600536810022087/im2211sup1.cif

Structure factors: contains datablocks I. DOI: 10.1107/S1600536810022087/im2211Isup2.hkl

Additional supplementary materials:  crystallographic information; 3D view; checkCIF report

## Figures and Tables

**Table 1:** Hydrogen-bond geometry (Å, °)

*D*—H⋯*A*	*D*—H	H⋯*A*	*D*⋯*A*	*D*—H⋯*A*
N1—H1⋯N7^i^	0.90	2.05	2.932 (3)	166

Symmetry code: (i) 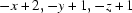.

**Fig. 1.**
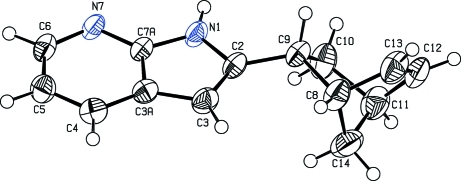
Molecular structure of compound I. Displacement ellipsoids are drawn at the 50% probability level. H atoms are depicted as circles of arbitrary size.

## supplementary crystallographic information

### Comment

The interest in 7- azaindoles as bioisoster of indole or purine has arisen in conjunction with recent pharmacological programs. Numerous publications on its derivatization reflect the increasing attention paid to this heterocyclic system. The crystal structure of 2-bicyclo[2.2.1]hept-5-en-2-yl-1*H*-pyrrolo[2,3-*b*]pyridine, C_14_H_14_N_2_, is characterized by an intermolecular hydrogen bond N1—H1··· N7 (2.05 Å) forming dimers of enantiomeric molecules, which are related by a crystallographic centre of symmetry.

### Experimental

3-methylpyridine (4 g, 43 mmol) was added dropwise to a freshly prepared solution of LDA in THF (0.9*M*) (59 ml, 53 mmol) at 273 K. The resulting suspension was stirred at 273 K for 30 min. Racemic 5-norbornene-2-carbonitrile (5.12 g, 43 mmol) was added dropwise at such a rate that the temperature did not rise above 283 K. Stirring was continued for 60 min. at 273 K. Another portion of LDA solution (59 ml, 53 mmol) was added and stirring was continued overnight at 333 K. The final reaction mixture was allowed to cool and ice-water was added. The mixture was extracted with ethylacetate and the combined extracts were dried (Na_2_SO_4_) and the solvent was evaporated under reduced pressure. The residue was subjected to flash chromatography. The title compound was obtained in a yield of 31% (2.834 g, 13.48 mmol). Crystals suitable for X-ray analysis were obtained by slow evaporation of the solvent from a methanolic solution.

### Refinement

Hydrogen atoms attached to carbons were placed at calculated positions with C—H = 0.95 Å (aromatic) or 0.98–0.99 Å (*sp*^3^ C-atom). All H atoms were refined in the riding-model approximation with isotropic displacement parameters (set at 1.2–1.5 times of the *U*_eq_ of the parent atom). The hydrogen atom attached to N1 was located in diff. Fourier maps and refined using a fixed isotropic displacement parameter and applying a riding motion model.

### Crystal data

**Table:**

C_14_H_14_N_2_	*F*(000) = 448
*M**_r_* = 210.27	*D*_x_ = 1.258 Mg m^−^^3^
Monoclinic, *P*2_1_/*n*	Cu *K*α radiation, λ = 1.54178 Å
Hall symbol: -P 2yn	Cell parameters from 25 reflections
*a* = 7.7837 (12) Å	θ = 65–69°
*b* = 8.9867 (14) Å	µ = 0.58 mm^−^^1^
*c* = 15.973 (3) Å	*T* = 193 K
β = 96.408 (8)°	Block, colourless
*V* = 1110.3 (3) Å^3^	0.40 × 0.30 × 0.20 mm
*Z* = 4	

### Data collection

**Table:**

Enraf–Nonius CAD-4 diffractometer	*R*_int_ = 0.050
Radiation source: rotating anode	θ_max_ = 70.0°, θ_min_ = 5.6°
graphite	*h* = −9→0
ω/2θ scans	*k* = −10→0
2274 measured reflections	*l* = −19→19
2113 independent reflections	3 standard reflections every 60 min
1940 reflections with *I* > 2σ(*I*)	intensity decay: 2%

### Refinement

**Table:**

Refinement on *F*^2^	Primary atom site location: structure-invariant direct methods
Least-squares matrix: full	Secondary atom site location: difference Fourier map
*R*[*F*^2^ > 2σ(*F*^2^)] = 0.082	Hydrogen site location: inferred from neighbouring sites
*wR*(*F*^2^) = 0.227	H-atom parameters constrained
*S* = 1.09	*w* = 1/[σ^2^(*F*_o_^2^) + (0.1178*P*)^2^ + 1.2641*P*] where *P* = (*F*_o_^2^ + 2*F*_c_^2^)/3
2113 reflections	(Δ/σ)_max_ < 0.001
145 parameters	Δρ_max_ = 0.47 e Å^−^^3^
0 restraints	Δρ_min_ = −0.36 e Å^−^^3^

### Special details

**Table:**

Geometry. All e.s.d.'s (except the e.s.d. in the dihedral angle between two l.s. planes) are estimated using the full covariance matrix. The cell e.s.d.'s are taken into account individually in the estimation of e.s.d.'s in distances, angles and torsion angles; correlations between e.s.d.'s in cell parameters are only used when they are defined by crystal symmetry. An approximate (isotropic) treatment of cell e.s.d.'s is used for estimating e.s.d.'s involving l.s. planes.
Refinement. Refinement of *F*^2^ against ALL reflections. The weighted *R*-factor *wR* and goodness of fit *S* are based on *F*^2^, conventional *R*-factors *R* are based on *F*, with *F* set to zero for negative *F*^2^. The threshold expression of *F*^2^ > σ(*F*^2^) is used only for calculating *R*-factors(gt) *etc*. and is not relevant to the choice of reflections for refinement. *R*-factors based on *F*^2^ are statistically about twice as large as those based on *F*, and *R*- factors based on ALL data will be even larger.

### Fractional atomic coordinates and isotropic or equivalent isotropic displacement parameters (Å^2^)

**Table:**

	*x*	*y*	*z*	*U*_iso_*/*U*_eq_	
N1	0.8046 (3)	0.5132 (3)	0.41997 (15)	0.0360 (6)	
H1	0.8691	0.5799	0.4519	0.043*	
C2	0.6512 (3)	0.5373 (3)	0.36871 (17)	0.0362 (6)	
C3	0.5981 (3)	0.4080 (3)	0.32996 (18)	0.0399 (7)	
H3	0.4964	0.3943	0.2920	0.048*	
C3A	0.7228 (3)	0.2965 (3)	0.35653 (16)	0.0345 (6)	
C4	0.7490 (4)	0.1469 (3)	0.33978 (17)	0.0391 (7)	
H4	0.6692	0.0929	0.3020	0.047*	
C5	0.8949 (4)	0.0794 (3)	0.37998 (18)	0.0411 (7)	
H5	0.9164	−0.0228	0.3700	0.049*	
C6	1.0105 (3)	0.1604 (3)	0.43497 (18)	0.0387 (7)	
H6	1.1092	0.1100	0.4615	0.046*	
N7	0.9921 (3)	0.3044 (3)	0.45291 (14)	0.0355 (6)	
C7A	0.8499 (3)	0.3677 (3)	0.41290 (16)	0.0317 (6)	
C8	0.3998 (4)	0.7013 (4)	0.3032 (2)	0.0472 (8)	
H8	0.3817	0.6311	0.2544	0.057*	
C9	0.5793 (3)	0.6919 (3)	0.35899 (18)	0.0383 (7)	
H9	0.6638	0.7548	0.3321	0.046*	
C10	0.5422 (5)	0.7683 (4)	0.4420 (2)	0.0528 (9)	
H10A	0.6201	0.8542	0.4552	0.063*	
H10B	0.5561	0.6974	0.4897	0.063*	
C11	0.3507 (4)	0.8199 (4)	0.4233 (2)	0.0545 (9)	
H11	0.2909	0.8473	0.4734	0.065*	
C12	0.3445 (4)	0.9316 (4)	0.3555 (2)	0.0485 (8)	
H12	0.3232	1.0350	0.3612	0.058*	
C13	0.3743 (4)	0.8614 (4)	0.2839 (2)	0.0532 (9)	
H13	0.3782	0.9068	0.2304	0.064*	
C14	0.2793 (4)	0.6819 (4)	0.3730 (2)	0.0573 (9)	
H14A	0.3002	0.5879	0.4049	0.069*	
H14B	0.1554	0.6913	0.3518	0.069*	

### Atomic displacement parameters (Å^2^)

**Table:**

	*U*^11^	*U*^22^	*U*^33^	*U*^12^	*U*^13^	*U*^23^
N1	0.0236 (10)	0.0376 (12)	0.0445 (12)	0.0054 (9)	−0.0063 (9)	−0.0044 (10)
C2	0.0249 (12)	0.0446 (15)	0.0376 (13)	0.0056 (11)	−0.0034 (10)	0.0010 (11)
C3	0.0298 (13)	0.0458 (16)	0.0417 (14)	0.0002 (11)	−0.0058 (11)	−0.0010 (12)
C3A	0.0302 (13)	0.0406 (14)	0.0325 (12)	−0.0005 (11)	0.0024 (10)	−0.0005 (11)
C4	0.0386 (14)	0.0425 (15)	0.0365 (14)	−0.0024 (12)	0.0052 (11)	−0.0048 (12)
C5	0.0428 (15)	0.0378 (15)	0.0440 (15)	0.0057 (12)	0.0109 (12)	−0.0055 (12)
C6	0.0319 (13)	0.0412 (15)	0.0436 (15)	0.0109 (11)	0.0070 (11)	−0.0003 (12)
N7	0.0236 (10)	0.0402 (12)	0.0422 (12)	0.0070 (9)	0.0013 (9)	−0.0024 (10)
C7A	0.0246 (12)	0.0362 (13)	0.0342 (13)	0.0042 (10)	0.0028 (10)	0.0004 (10)
C8	0.0381 (15)	0.0533 (18)	0.0484 (17)	0.0099 (13)	−0.0028 (13)	−0.0038 (14)
C9	0.0264 (13)	0.0452 (16)	0.0424 (14)	0.0059 (11)	0.0006 (11)	0.0011 (12)
C10	0.0521 (19)	0.059 (2)	0.0451 (16)	0.0176 (15)	−0.0032 (14)	−0.0003 (15)
C11	0.0504 (19)	0.064 (2)	0.0525 (18)	0.0156 (16)	0.0187 (15)	0.0002 (16)
C12	0.0328 (14)	0.0534 (18)	0.0593 (19)	0.0152 (13)	0.0044 (13)	−0.0024 (14)
C13	0.0432 (17)	0.065 (2)	0.0508 (17)	0.0135 (15)	0.0010 (14)	0.0083 (16)
C14	0.0337 (16)	0.068 (2)	0.072 (2)	−0.0002 (15)	0.0140 (15)	−0.0068 (18)

### Geometric parameters (Å, °)

**Table:**

N1—C7A	1.362 (3)	C8—C14	1.546 (5)
N1—C2	1.388 (3)	C8—C9	1.573 (4)
N1—H1	0.9027	C8—H8	1.0000
C2—C3	1.359 (4)	C9—C10	1.548 (4)
C2—C9	1.500 (4)	C9—H9	1.0000
C3—C3A	1.426 (4)	C10—C11	1.558 (5)
C3—H3	0.9500	C10—H10A	0.9900
C3A—C4	1.391 (4)	C10—H10B	0.9900
C3A—C7A	1.414 (4)	C11—C12	1.473 (5)
C4—C5	1.381 (4)	C11—C14	1.547 (5)
C4—H4	0.9500	C11—H11	1.0000
C5—C6	1.391 (4)	C12—C13	1.349 (5)
C5—H5	0.9500	C12—H12	0.9500
C6—N7	1.336 (4)	C13—H13	0.9500
C6—H6	0.9500	C14—H14A	0.9900
N7—C7A	1.342 (3)	C14—H14B	0.9900
C8—C13	1.480 (5)		
			
C7A—N1—C2	108.4 (2)	C2—C9—C10	115.2 (2)
C7A—N1—H1	123.5	C2—C9—C8	114.0 (2)
C2—N1—H1	128.1	C10—C9—C8	102.9 (2)
C3—C2—N1	109.4 (2)	C2—C9—H9	108.2
C3—C2—C9	130.9 (2)	C10—C9—H9	108.2
N1—C2—C9	119.5 (2)	C8—C9—H9	108.2
C2—C3—C3A	107.6 (2)	C9—C10—C11	103.5 (2)
C2—C3—H3	126.2	C9—C10—H10A	111.1
C3A—C3—H3	126.2	C11—C10—H10A	111.1
C4—C3A—C7A	116.9 (2)	C9—C10—H10B	111.1
C4—C3A—C3	137.0 (3)	C11—C10—H10B	111.1
C7A—C3A—C3	106.0 (2)	H10A—C10—H10B	109.0
C5—C4—C3A	117.8 (3)	C12—C11—C14	100.6 (3)
C5—C4—H4	121.1	C12—C11—C10	107.2 (3)
C3A—C4—H4	121.1	C14—C11—C10	98.1 (3)
C4—C5—C6	120.3 (3)	C12—C11—H11	116.1
C4—C5—H5	119.9	C14—C11—H11	116.1
C6—C5—H5	119.9	C10—C11—H11	116.1
N7—C6—C5	124.4 (3)	C13—C12—C11	108.0 (3)
N7—C6—H6	117.8	C13—C12—H12	126.0
C5—C6—H6	117.8	C11—C12—H12	126.0
C6—N7—C7A	114.3 (2)	C12—C13—C8	108.1 (3)
N7—C7A—N1	125.1 (2)	C12—C13—H13	126.0
N7—C7A—C3A	126.3 (2)	C8—C13—H13	126.0
N1—C7A—C3A	108.6 (2)	C8—C14—C11	94.1 (3)
C13—C8—C14	100.5 (3)	C8—C14—H14A	112.9
C13—C8—C9	105.1 (3)	C11—C14—H14A	112.9
C14—C8—C9	99.0 (2)	C8—C14—H14B	112.9
C13—C8—H8	116.5	C11—C14—H14B	112.9
C14—C8—H8	116.5	H14A—C14—H14B	110.3
C9—C8—H8	116.5		
			
C7A—N1—C2—C3	0.6 (3)	N1—C2—C9—C10	−58.2 (4)
C7A—N1—C2—C9	−175.0 (2)	C3—C2—C9—C8	8.7 (4)
N1—C2—C3—C3A	−0.6 (3)	N1—C2—C9—C8	−176.8 (2)
C9—C2—C3—C3A	174.4 (3)	C13—C8—C9—C2	−166.1 (3)
C2—C3—C3A—C4	−177.8 (3)	C14—C8—C9—C2	90.4 (3)
C2—C3—C3A—C7A	0.3 (3)	C13—C8—C9—C10	68.6 (3)
C7A—C3A—C4—C5	0.8 (4)	C14—C8—C9—C10	−35.0 (3)
C3—C3A—C4—C5	178.8 (3)	C2—C9—C10—C11	−127.4 (3)
C3A—C4—C5—C6	−0.2 (4)	C8—C9—C10—C11	−2.8 (3)
C4—C5—C6—N7	−0.2 (4)	C9—C10—C11—C12	−64.3 (3)
C5—C6—N7—C7A	−0.1 (4)	C9—C10—C11—C14	39.5 (3)
C6—N7—C7A—N1	−179.0 (2)	C14—C11—C12—C13	−32.7 (3)
C6—N7—C7A—C3A	0.8 (4)	C10—C11—C12—C13	69.4 (4)
C2—N1—C7A—N7	179.4 (2)	C11—C12—C13—C8	0.3 (4)
C2—N1—C7A—C3A	−0.4 (3)	C14—C8—C13—C12	32.2 (3)
C4—C3A—C7A—N7	−1.1 (4)	C9—C8—C13—C12	−70.2 (3)
C3—C3A—C7A—N7	−179.7 (3)	C13—C8—C14—C11	−48.3 (3)
C4—C3A—C7A—N1	178.6 (2)	C9—C8—C14—C11	59.0 (3)
C3—C3A—C7A—N1	0.1 (3)	C12—C11—C14—C8	48.6 (3)
C3—C2—C9—C10	127.3 (3)	C10—C11—C14—C8	−60.7 (3)

### Hydrogen-bond geometry (Å, °)

**Table:**

*D*—H···*A*	*D*—H	H···*A*	*D*···*A*	*D*—H···*A*
N1—H1···N7^i^	0.90	2.05	2.932 (3)	166

Symmetry codes: (i) −*x*+2, −*y*+1, −*z*+1.
